# Case report: B7-H3 CAR-T therapy partially controls tumor growth in a basal cell carcinoma patient

**DOI:** 10.3389/fonc.2022.956593

**Published:** 2022-08-17

**Authors:** Gang Hu, Guangchao Li, Wei Wen, Wen Ding, Zhao Zhou, Yongwei Zheng, Taoyuan Huang, Junnan Ren, Rongyi Chen, Dingheng Zhu, Renliang He, Yunsheng Liang, Min Luo

**Affiliations:** ^1^ Department of Dermatology, Dermatology Hospital, Southern Medical University, Guangzhou, China; ^2^ Research and Development Department Guangzhou Bio-Gene Technology Co., Ltd., Guangzhou, China

**Keywords:** basal cell carcinomas, skin cancers, CAR-T cell therapy, B7-H3, CD276, intratumoral injection

## Abstract

B7-H3 is over-expressed in multiple types of solid tumors, making it an ideal target for chimeric antigen receptor (CAR)-T therapy. Here, we first report a case of multiple basal cell carcinoma (BCC) patient treated with humanized monoclonal anti-B7-H3 CAR-T cells through direct intratumoral injection. After three dose-escalated injections, the lesion in the abdomen decreased by 40% in volume, shrank from bulging to flat, but was not eradicated completely. The large lesion in the forehead became dry from original ulcer and bleeding. The adverse events observed were itching, myalgia, and redness. Immunohistochemistry analysis demonstrated that B7-H3-positive tumor cells and B7-H3 expression intensity were reduced after injections of CAR-T cells. The number of infiltrating CD3 T cells increased significantly but mainly located outside the tumor region. Subsequently, high levels of TGF-β in the tumor area were observed, suggesting that solid tumor microenvironment may hinder the infiltration and effect of CAR-T cells. In summary, in this particular case report, intratumoral injection of B7-H3 CAR-T cells partially controls tumor growth in the BCC patient with minor adverse events. The efficacy and safety of B7-H3 CAR-T therapy need to be further investigated with a larger cohort of patients. Although only one clinical case is reported here, the anti-B7-H3 CAR-T cell therapy should be considered as a treatment option for solid tumors in the future. This clinical trial was registered at the Chinese Clinical Trial Registry (www.chictr.org.cn) with registration number ChiCTR2100044386.

## Background

Chimeric antigen receptor (CAR)-T cell therapy has achieved remarkable success for hematopoietic malignancies, including CD19 CAR-T for B-cell acute lymphoblastic leukemia (B-ALL) and BCMA CAR-T for multiple myeloma (MM) (1, 2). However, the same success has not been replicated for solid tumors (3). One of the challenges is to identify a CAR-T target specifically expressed on solid tumors, but not on normal tissues.

B7-H3 (also known as CD276) is an immune checkpoint molecule, with no or low expression on normal tissues, but with high expression on glioblastoma, ovarian cancer, non-small-cell lung cancer, squamous cell carcinomas, melanoma, and other malignant solid tumors. B7-H3 has been shown to contribute to tumor metastasis (4–6) and is generally associated with poor clinical prognosis. Based on these findings, B7-H3 has been considered as a promising target for cancer immunotherapy. B7-H3-targeted monoclonal antibody and its derivative forms with drug conjugation or with dual specificity were all being studied and have entered clinical stage. In addition, several studies have demonstrated that B7-H3-targeted CAR-T cells have potent preclinical activity in pediatric and adult solid tumors, including neuroblastoma, rhabdoid tumors, and other types of solid tumors (7–9).

Skin cancer is a commonly examined tumor model in the context of immunotherapy, since it is known for its immunogenic features. B7-H3 has been shown to be highly expressed on 90% of skin cancers (10, 11), including melanoma, basal cell carcinoma (BCC), and squamous cell carcinoma (SCC) (**Supplementary Figure 1**). Among them, BCC is the most prevalent pathological type of skin cancer (12, 13). For primary BCCs, standard surgical excision is often an appropriate treatment option. Although BCCs usually progress indolently, a small subset of BCCs can develop into metastatic or local advanced BCCs (LaBCCs) through local invasion (12, 14). For LaBCCs, and recurrent or unresectable BCCs, systemic therapy is necessary. However, treatment-emergent adverse events often lead to discontinuation of therapy (15); therefore, new therapeutic strategies need to be developed. Immunotherapy has become a powerful clinical strategy for treating malignant tumors (16). For treatment of BCCs, especially LaBCCs, immunotherapy is undoubtedly a highly promising choice, such as using PD-1 blockade (17, 18). Although the clinical utility of CAR-T in solid tumors is still relatively limited, a large number of studies have begun to address the efficacy and safety issues of its clinical application (3, 19–22). To our knowledge, there has yet been no report of CAR-T-based immunotherapy on BCCs.

In an effort to bring the powerful CAR-T therapy into skin cancer, we applied autologous B7-H3-CAR-T cells as a novel immunotherapy for BCCs. Here, we report a case of multiple BCC patient who has relapsed after multiple surgeries and therapies over the past 10 years. After three doses of autologous anti-B7-H3 CAR-T cells through intratumoral injection, the patient achieved partial response and had temporary and mild side effects. This study suggested a potential therapeutic strategy for patients with B7-H3-positive malignant solid tumors.

## Case presentation

**Figure 1:**
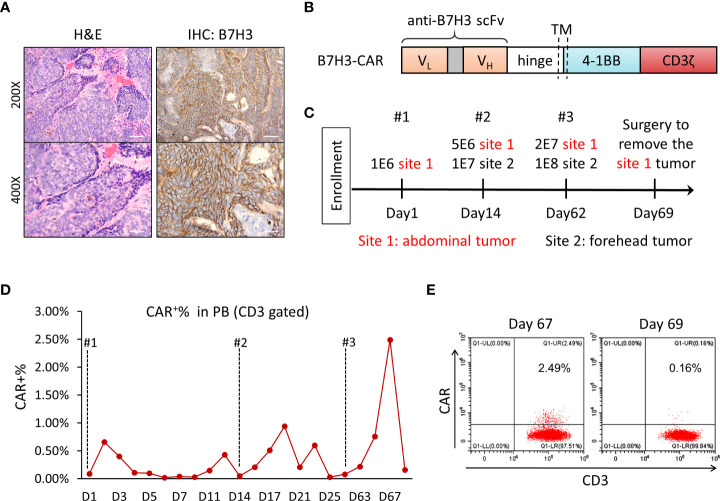
Clinical examinations in the sequential injection of B7-H3 CAR-T cell therapy. **(A)** Histological analysis of basal cell carcinoma sections by hematoxylin and eosin (H&E) and anti-B7-H3 staining for diagnosis. Slides were analyzed and scored by two individual pathologists independently. **(B)** Schematic diagram of B7-H3 CAR vector. V_L_, variable L chain; V_H_, variable H chain; TM, transmembrane domain. **(C)** The protocol of B7-H3 CAR-T injection. Intratumoral injection was performed at two tumor sites for a total of three times, on days 1, 14, and 62. **(D)** Detection of CAR-T (CD3^+^CAR^+^) cells in peripheral blood during immunotherapy. **(E)** Flow cytometry analysis of CD3^+^CAR^+^ cells in peripheral blood on days 67 and 69 after CAR-T cell injection. FITC-labeled recombinant B7-H3 protein was used to detect B7-H3-CAR transduced CD3^+^ cells.

A 65-year-old man was initially diagnosed with multiple BCC in 2003, skin examination showed multiple dark-colored plaques or papules on the patient’s forehead and abdomen, and the larger plaques showed ulceration and bleeding. Histopathology of the abnormal skin tissue showed palisade-like basophilic basaloid epithelium with cleft forming from adjacent stroma. The patient had received multiple lines of chemotherapy and surgery thereafter. However, the disease relapsed and spread to all over the body. Some lesions, especially the large one (6 cm × 6 cm) in the forehead, were accompanied by ulcer exudate and pain, and were not removable by surgery. The patient requested for substitutive therapy options. Immunohistochemical staining of the tumor tissue showed positive for B7-H3, with 80% positivity and ++/+++ intensity (**Figure 1A**); thus, the patient was accepted to enroll in the clinical trial of B7-H3-CAR-T immunotherapy. The patient signed the informed consent before starting apheresis. Given that this was our first attempt to treat the patient with anti-B7-H3 CAR-T cells for the clearance of skin tumors, we performed intratumoral injection instead of intravenous injection, with the aim to increase the quantity and concentration of CAR-T cells in the local tumor area. Three doses of CAR-T injection were performed. The first intratumoral injection of CAR-T was administered in November 2020, the second one in December 2020, and the third one in January 2021.

A humanized single-chain variable fragment (scFv) against B7-H3 (Patent No. ZL 2020 1 0622482.X) was used to obtain the 4-1BB-based second-generation CAR-T (**Figure 1B**). The tumor-killing capacity of B7-H3-CAR-T cells was tested *in vitro* and *in vivo*; all results indicated the promising potential of CAR-T cells in killing B7-H3-positive tumor cells and controlling tumor growth in the tumor-bearing NSG mouse model (**Supplementary Figure 2**).

In this particular study, no lymphodepleting chemotherapy was given to the patient before CAR-T cell injection in order to keep the immune system intact. In this course of CAR-T therapy, the patient received autologous anti-B7-H3 CAR-T cells through intratumoral injection three times with dose escalation on day 1, day 14, and day 62 (**Figure 1C**). There were two different tumor sites that were treated in this patient (site 1 for abdominal tumor 1.8 cm × 0.6 cm; site 2 for forehead tumor 6 cm × 6 cm). Two batches of CAR-T cells were prepared for injections, CAR transduction rate was 52.50% and 42.16%, and the CD4/CD8 ratio was 3.25 and 1.89, respectively (**Supplementary Figure 3**). The expansion and percentage of CAR-positive T cells in peripheral blood after injection were analyzed by flow cytometry using FITC-labeled recombinant B7-H3 protein. Very low levels of CAR-T cells were detected in peripheral blood throughout the treatment. Only 2.5% of total T cells in peripheral blood was CAR positive at day 5 after the third injection (**Figure 1D**), and the percentage of CAR-T cells was decreased to undetectable levels 2 days later (**Figures 1D, E**).

The patient’s alanine transaminase (ALT) and aspartate transaminase (AST) levels were slightly increased about twofold after the first injection (**Supplementary Figure 4A**). After reaching the peak on day 6, the levels of ALT and AST began to slowly decline. However, we did not observe increased ALT and AST levels after the second and third injection (**Supplementary Figure 4A**). The plasma levels of γ-glutamyl transpeptidase (γ-GT) and alkaline phosphatase (ALP) of the patient were relatively stable during the treatment period (**Supplementary Figure 4B**). Meanwhile, the plasma IL-6 stayed at lower levels during CAR-T treatment (**Supplementary Figure 4C**). The patient’s body weight was stable over the treatment period (data not shown), without any treatment-related significant changes. There was no obvious abnormality in blood routine tests during CAR-T treatment, including cell counts of white blood cells (WBCs), neutrophils, platelets, lymphocytes, monocytes, and the concentration of hemoglobin (**Supplementary Figure 5**).

**Figure 2:**
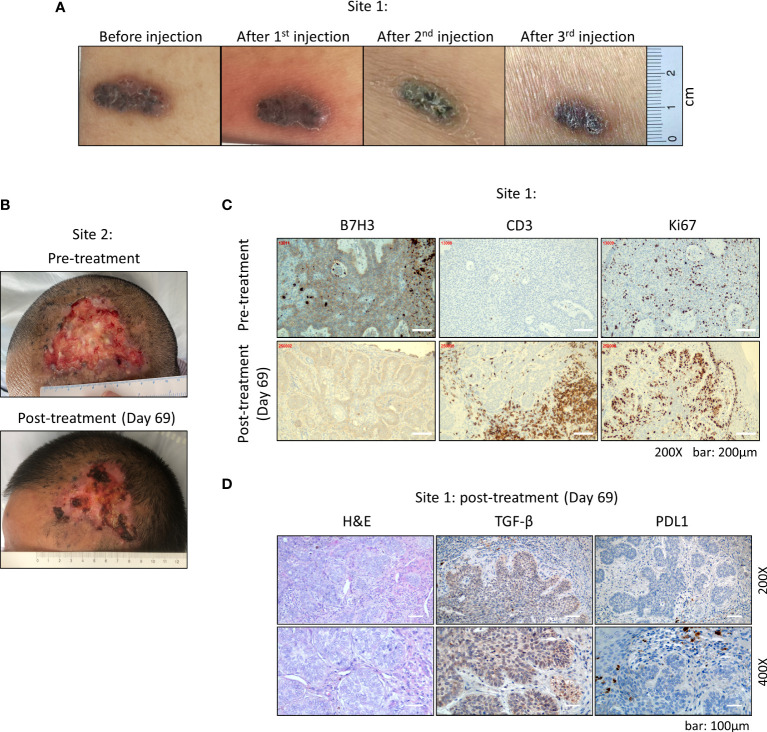
Clinical manifestation of multiple basal cell carcinoma after CAR-T cell therapy. **(A)** Abdominal tumor at site 1. After each injection, the tumor gradually shrank in size. **(B)** The large lesion of forehead tumor at site 2 became dry from original ulcer and bleeding. **(C)** Staining of B7-H3, CD3, and Ki67 of abdominal tumor at site 1 by immunohistochemistry (IHC). B7-H3^+^ cells showed sub-epithelial localization and were widely observed within the tumor tissue. CD3 staining showed an enrichment of T cells at the injection site within the tumor. **(D)** Expressions of TGF-β and PDL1 within the tumor tissue examined by IHC. All IHC slides were analyzed and scored by two individual pathologists independently.

After three doses of injections, the volume of original lesion in the abdomen (site 1) was decreased by 40% (from 1.8 cm × 0.6 cm to 1.3 cm × 0.5 cm) in size, shrank from bulging to flat (**Figure 2A**). The large lesion in the forehead (site 2) became dry from original ulcer and bleeding (**Figure 2B**). We further performed surgery to remove the remaining tumor in the abdomen after the third dose of CAR-T injection. IHC analysis indicated that the percentage of B7-H3-positive cells was decreased from 80% to about 40%, and the B7-H3 intensity was decreased from ++/+++ to + after the third injection of CAR-T cells (**Figure 2C**). Meanwhile, CD3 T cells were enriched in the injection site within the tumor; however, majority of these T cells failed to infiltrate into neighboring tumor area, which was stained positive for B7-H3 and Ki67 (**Figure 2C**). Subsequently, the expressions of two common inhibitory signaling molecules TGF-β and PD-L1 were examined, and high expression level of TGF-β was observed in the tumor area upon CAR-T treatment (**Figure 2D**).

Eventually, tumor growth on both sites 1 and 2 was partially controlled after B7-H3 CAR-T treatment; however, the tumors were not entirely eradicated. In the follow-up visits, the patient was under stable physiological condition and showed no sequela upon CAR-T injections, and was continuously given with the routine non-surgical treatments on the tumorous skin.

## Discussion

CAR-T technology targets a specific antigen rather than a single cancer disease; thus, the selection of targets is fundamental to the efficacy and safety of CAR-T therapy. In this case, we reported for the first time that a patient with skin tumors achieved a partial response after receiving repeated intratumoral injections of anti-B7-H3 CAR-T cells.

While B7-H3 protein is expressed at low levels in most of the normal tissues, it is aberrantly expressed on differentiated malignant cells and cancer-initiating cells, with limited heterogeneity, and in multiple tumor types, including lung, colon, breast, and ovarian cancers (23). Hence, B7-H3 has been proposed as an attractive target for CAR-T cell therapy. Recently, several CAR-T therapies targeting B7-H3 have generated promising preclinical results against hematologic and multiple solid tumors (7–9, 24–30), including acute myeloid leukemia, melanoma, natural killer/T-cell lymphoma, brain tumors, atypical teratoid/rhabdoid tumors, glioblastoma, neuroblastoma, anaplastic meningioma, and non-small cell lung cancer (NSCLC). Specifically, recent developed anti-B7-H3 CAR-T inhibited the growth of different B7-H3-positive melanoma xenografts in preclinical studies (9, 24).

In this study, after each injection, the patient reported mild subjective myalgia, which resolved within 48 h. This patient’s subjective symptoms were not attributable to IL-6 release (**Supplementary Figure 4**). In addition, the patient developed itchy and redness at the injection sites, which might be related to the local responses of CAR-T cells. Notably, patient’s serum levels of ALT and AST were slightly increased about twofold after the first injection of CAR-T cells, and then both ALT and AST levels began to decline and returned to normal range. However, no more increase of ALT and AST levels were observed after the second and third injections, even with much higher dosage of CAR-T cells (**Figure 1C**), indicating that the increased ALT and AST serum levels may not be related to the CAR-T therapy.

Similar to the previous study (31), a very low percentage (<3%) of CAR-positive T cells was detected in the circulating peripheral blood after intratumoral injection (**Figure 1D**), suggesting that extravasation of anti-B7-H3 CAR-T cells had occurred. However, unlike the surge in CAR-T cells when treating hematologic malignancies, the anti-B7-H3 CAR-T cells in the peripheral blood rapidly decreased over the next 2 days (**Figure 1E**), which may also indicate the restricted expression of B7-H3 in normal tissues. Given no serious adverse effects were observed in the patient, we postulated that systemic injection of anti-B7-H3 CAR T cells might be tolerated.

We also found that the expression of B7-H3 on tumors was decreased after three doses of injections (**Figure 2C**), indicating that tumor cells with high expression of B7-H3 were eliminated preferentially. A group of infiltrating CD3^+^ T cells in tumor tissue was found at the injection site on day 7 after the third intratumoral injection. However, we cannot distinguish the origin of these T cells since no lymphodepletion conditioning was performed on the patient before CAR-T treatment (**Figure 2C**). Nevertheless, these T cells appeared to be concentrated around the injection sites only, and excluded from the neighboring tumor area. We assumed two reasons to explain this phenomenon. First, injected CAR-T cells have cleared the local B7-H3-positive tumor cells at the injection site; however, they failed to migrate into the adjacent tumor region. Second, as shown in **Figure 2D**, high level of TGF-β and scattered expression of PD-L1 were observed within the tumor area, and these immune inhibitory molecules suppressed the infiltration of CAR-T cells into the neighboring tumor area. Altogether, these observations suggested that solid tumor microenvironment might deter CAR-T cell infiltration and function.

Based on the current case report, anti-B7-H3 CAR-T cell treatment exhibited promising potential in treating skin cancers, although the patient in this study achieved partial response after three doses of intratumoral injections. To further evaluate the efficacy and safety of anti-B7-H3 CAR-T cell treatment on skin cancers, a larger cohort study is warranted.

## Conclusions

In conclusion, one patient with basal cell carcinoma received anti-B7-H3 CAR-T treatment, and the tumor growth was partially controlled after three doses of intratumoral injections; meanwhile, minor and manageable side effects were observed. These clinical outcomes might indicate the great efficacy of anti-B7-H3 CAR-T in treating skin cancers, although these data were obtained from only one patient. A larger cohort study to further evaluate the efficacy and safety issues of anti-B7-H3 CAR-T therapy is required in the near future.

## Data availability statement

The original contributions presented in the study are included in the article/**Supplementary Material**. Further inquiries can be directed to the corresponding authors.

## Ethics statement

The study protocol was approved by the institutional review board of Dermatology Hospital, Southern Medical University. The study was performed in compliance with Good Clinical Practice guidelines. Written informed consent was obtained from the patient. The IRB approval number is 2020009.

## Author contributions

GH, GL, YL, and ML designed the study. Acquisition of data and analysis were done by WW, WD, and ZZ. Analysis and interpretation of data were done by GL, TH, JR, and RC. Writing and revision of the manuscript were done by GH, GL, YZ, ML, RH, and DZ. All authors contributed to the article and approved the submitted version.

## Funding

This work was supported by the Guangzhou Science and Technology Project (202206010141) and the Natural Science Foundation of Xinjiang Uygur Autonomous Region Youth Project (2021D01C351), and partially funded by the Pearl River S&T Nova Program of Guangzhou (201906010056 for GL).

## Acknowledgments

The authors would like to thank the patient who participated in the trial and the patient’s family for their support. The authors would also like to thank the physicians, nurses, research coordinators, and other staff at the hospital and the R&D center who assisted with the study. The authors would also like to thank Anbiping Biotech Co., for doing the immunostaining of tumor samples.

## Conflict of interest

Authors GL, WD, ZZ, YZ and ML were employed by the company Guangzhou Bio-Gene Technology Co., Ltd. 

The remaining authors declare that the research was conducted in the absence of any commercial or financial relationships that could be construed as a potential conflict of interest.

## Publisher’s note

All claims expressed in this article are solely those of the authors and do not necessarily represent those of their affiliated organizations, or those of the publisher, the editors and the reviewers. Any product that may be evaluated in this article, or claim that may be made by its manufacturer, is not guaranteed or endorsed by the publisher.

## Supplementary material

Supplementary Figure 1 Immunohistochemistry detection of B7-H3 expression in skin tumors. **(A)** B7-H3 expression in malignant melanoma with skin tissue array (DC-MaL11011; 24 cases/24 cores). **(B)** High expression levels of B7-H3 were observed in melanoma, basal cell carcinoma (BCC), and squamous cell carcinoma (SCC). All IHC slides were analyzed and scored by two individual pathologists independently.

Supplementary Figure 2 B7-H3-CAR-T exhibited excellent antitumor efficacy *in vitro* and *in vivo*. **(A)** B7-H3-CAR-T was effective in killing a variety of solid tumor cells *in vitro* in a dose-dependent manner. PL45, human pancreatic cancer cells; PC9, human non-small cell lung cancer cells; HepG2, human hepatocellular carcinoma cells; SiHa, human cervical cancer cells. **(B)** B7-H3-CAR-T effectively cleared transplanted tumors in immuno-deficient NSG mice. Tumor cells (Hep3B, A375 and SiHa) carrying luciferase gene were inoculated subcutaneously on the back of mice for more than 10 days, and the mice were then injected intravenously with B7-H3-CAR-T cells (3×10^6^ cells/mouse were given in mice bearing Hep3B and A375, three therapeutic doses were given in SiHa transplanted mice). **(C)** Tumor volumes were measured with a vernier caliper, and the results showed that B7-H3 CAR-T completely eliminated the transplanted tumors in mice.

Supplementary Figure 3 Characterizations of the B7-H3-CAR-T cells. **(A)** The table shows the identification results of two batches of B7-H3-CAR-T cells. CAR-T cell activity was determined by Smart Cell Counter NucleoCounter^®^ NC-200TM (ChemoMetec, Danmark). 200 µL suspended cells was added into Vial-CassetteTM containing dried acridine orange (AO) and DAPI dyes. Dead cells were dyed with AO/PI and counted by NucleoCounter. The cell activity was presented as (total cell number - dead cell number)/total cell number × 100%. A single copy gene (*RPPH1*)-based duplex qPCR assay was used to determine vector copy numbers (VCNs) in CAR T cell products. **(B)** Percentages of CD3, CD4, CD8, CAR positive T cells were determined by flow cytometry analysis.

Supplementary Figure 4 Monitoring of liver damage and IL6 levels in blood during immunotherapy. **(A, B)** The levels of alanine transaminase (ALT), aspartate transaminase (AST), alkaline phosphatase (ALP), and gamma-glutamyl transferase (γ-GT) were examined to evaluate the liver function after CAR-T cell injections. **(C)** Levels of IL-6 remained low after CAR-T cell injections.

Supplementary Figure 5 Measurement of blood components before and after the CAR-T treatment.

## References

[1] Raje N Berdeja J Lin Y Siegel D Jagannath S Madduri D. Anti-BCMA CAR T-cell therapy bb2121 in relapsed or refractory multiple myeloma. N Engl J Med (2019) 380:1726–37. doi: 10.1056/NEJMoa1817226 PMC8202968 31042825

[2] Maude SL Laetsch TW Buechner J Rives S Boyer M Bittencourt H. Tisagenlecleucel in children and young adults with b-cell lymphoblastic leukemia. N Engl J Med (2018) 378:439–48. doi: 10.1056/NEJMoa1709866 PMC5996391 29385370

[3] Martinez M Moon EK. CAR T cells for solid tumors: New strategies for finding, infiltrating, and surviving in the tumor microenvironment. Front Immunol (2019) 10:128. doi: 10.3389/fimmu.2019.00128 30804938 PMC6370640

[4] Yang S Wei W Zhao Q. B7-H3, a checkpoint molecule, as a target for cancer immunotherapy. Int J Biol Sci (2020) 16:1767–73. doi: 10.7150/ijbs.41105 PMC7211166 32398947

[5] Tekle C Nygren MK Chen YW Dybsjord I Nesland JM Maelandsmo GM. B7-H3 contributes to the metastatic capacity of melanoma cells by modulation of known metastasis-associated genes. Int J Cancer (2012) 130:2282–90. doi: 10.1002/ijc.26238 21671471

[6] Dong P Xiong Y Yue J Hanley SJB Watari H. B7H3 as a promoter of metastasis and promising therapeutic target. Front Oncol (2018) 8:264. doi: 10.3389/fonc.2018.00264 30035102 PMC6043641

[7] Theruvath J Sotillo E Mount CW Graef CM Delaidelli A Heitzeneder S. Locoregionally administered B7-H3-targeted CAR T cells for treatment of atypical teratoid/rhabdoid tumors. Nat Med (2020) 26:712–9. doi: 10.1038/s41591-020-0821-8 PMC7992505 32341579

[8] Majzner RG Theruvath JL Nellan A Heitzeneder S Cui Y Mount CW. CAR T cells targeting B7-H3, a pan-cancer antigen, demonstrate potent preclinical activity against pediatric solid tumors and brain tumors. Clin Cancer Res (2019) 25:2560–74. doi: 10.1158/1078-0432.CCR-18-0432 PMC8456711 30655315

[9] Yang M Tang X Zhang Z Gu L Wei H Zhao S. Tandem CAR-T cells targeting CD70 and B7-H3 exhibit potent preclinical activity against multiple solid tumors. Theranostics (2020) 10:7622–34. doi: 10.7150/thno.43991 PMC7359081 32685008

[10] Flem-Karlsen K Tekle C Andersson Y Flatmark K Fodstad O Nunes-Xavier CE. Immunoregulatory protein B7-H3 promotes growth and decreases sensitivity to therapy in metastatic melanoma cells. Pigment Cell Melanoma Res (2017) 30:467–76. doi: 10.1111/pcmr.12599 28513992

[11] Varki V Ioffe OB Bentzen SM Heath J Cellini A Feliciano J. PD-L1, B7-H3, and PD-1 expression in immunocompetent vs. immunosuppressed patients with cutaneous squamous cell carcinoma. Cancer Immunol Immunother (2018) 67:805–14. doi: 10.1007/s00262-018-2138-8 PMC11028243 29484464

[12] Dika E Scarfi F Ferracin M Broseghini E Marcelli E Bortolani B. Basal cell carcinoma: A comprehensive review. Int J Mol Sci (2020) 21(15):5572. doi: 10.3390/ijms21155572 PMC7432343 32759706

[13] Cameron MC Lee E Hibler BP Barker CA Mori S Cordova M. Basal cell carcinoma: Epidemiology; pathophysiology; clinical and histological subtypes; and disease associations. J Am Acad Dermatol (2019) 80:303–17. doi: 10.1016/j.jaad.2018.03.060 29782900

[14] Ozgediz D Smith E Zheng J Otero J Tabatabai ZL Corvera CU. Basal cell carcinoma does metastasize. Dermatol Online J (2008) 14(8):5. 19061565

[15] Proctor AE Thompson LA O’Bryant CL. Vismodegib: an inhibitor of the hedgehog signaling pathway in the treatment of basal cell carcinoma. Ann Pharmacother (2014) 48:99–106. doi: 10.1177/1060028013506696 24259609

[16] Riley RS June CH Langer R Mitchell MJ. Delivery technologies for cancer immunotherapy. Nat Rev Drug Discov (2019) 18(3):175–96. doi: 10.1038/s41573-018-0006-z PMC6410566 30622344

[17] Yost KE Satpathy AT Wells DK Qi Y Wang C Kageyama R. Clonal replacement of tumor-specific T cells following PD-1 blockade. Nat Med (2019) 25:1251–9. doi: 10.1038/s41591-019-0522-3 PMC6689255 31359002

[18] Sabbatino F Marra A Liguori L Scognamiglio G Fusciello C Botti G. Resistance to anti-PD-1-based immunotherapy in basal cell carcinoma: A case report and review of the literature. J Immunother Cancer (2018) 6:126. doi: 10.1186/s40425-018-0439-2 30458852 PMC6247622

[19] Ma S Li X Wang X Cheng L Li Z Zhang C. Current progress in CAR-T cell therapy for solid tumors. Int J Biol Sci (2019) 15:2548–60. doi: 10.7150/ijbs.34213 PMC6854376 31754328

[20] Grosser R Cherkassky L Chintala N Adusumilli PS. Combination immunotherapy with CAR T cells and checkpoint blockade for the treatment of solid tumors. Cancer Cell (2019) 36:471–82. doi: 10.1016/j.ccell.2019.09.006 PMC7171534 31715131

[21] Ma L Dichwalkar T Chang JYH Cossette B Garafola D Zhang AQ. Enhanced CAR-T cell activity against solid tumors by vaccine boosting through the chimeric receptor. Science (2019) 365:162–8. doi: 10.1126/science.aav8692 PMC6800571 31296767

[22] Bagley SJ O’Rourke DM. Clinical investigation of CAR T cells for solid tumors: Lessons learned and future directions. Pharmacol Ther (2020) 205:107419. doi: 10.1016/j.pharmthera.2019.107419 31629009

[23] Seaman S Zhu Z Saha S Zhang XM Yang MY Hilton MB. Eradication of tumors through simultaneous ablation of CD276/B7-H3-Positive tumor cells and tumor vasculature. Cancer Cell (2017) 31:501–515 e508. doi: 10.1016/j.ccell.2017.03.005 28399408 PMC5458750

[24] Zhang Z Jiang C Liu Z Yang M Tang X Wang Y. B7-H3-Targeted CAR-T cells exhibit potent antitumor effects on hematologic and solid tumors. Mol Ther Oncolytics (2020) 17:180–9. doi: 10.1016/j.omto.2020.03.019 PMC7178328 32346608

[25] Lichtman EI Du H Shou P Song F Suzuki K Ahn S. Preclinical evaluation of B7-H3-specific chimeric antigen receptor T cells for the treatment of acute myeloid leukemia. Clin Cancer Res (2021) 27:3141–53. doi: 10.1158/1078-0432.CCR-20-2540 PMC8248479 33531429

[26] Zheng M Yu L Hu J Zhang Z Wang H Lu D. Efficacy of B7-H3-Redirected BiTE and CAR-T immunotherapies against extranodal nasal natural Killer/T cell lymphoma. Transl Oncol (2020) 13:100770. doi: 10.1016/j.tranon.2020.100770 32298986 PMC7160598

[27] Tang X Zhao S Zhang Y Wang Y Zhang Z Yang M. B7-H3 as a novel CAR-T therapeutic target for glioblastoma. Mol Ther Oncolytics (2019) 14:279–87. doi: 10.1016/j.omto.2019.07.002 PMC6713854 31485480

[28] Du H Hirabayashi K Ahn S Kren NP Montgomery SA Wang X. Antitumor responses in the absence of toxicity in solid tumors by targeting B7-H3 *via* chimeric antigen receptor T cells. Cancer Cell (2019) 35:221–237 e228. doi: 10.1016/j.ccell.2019.01.002 30753824 PMC6645919

[29] Tang X Liu F Liu Z Cao Y Zhang Z Wang Y. Bioactivity and safety of B7-H3-targeted chimeric antigen receptor T cells against anaplastic meningioma. Clin Transl Immunol (2020) 9:e1137. doi: 10.1002/cti2.1137 PMC7292833 32547742

[30] Liu J Yang S Cao B Zhou G Zhang F Wang Y. Targeting B7-H3 *via* chimeric antigen receptor T cells and bispecific killer cell engagers augments antitumor response of cytotoxic lymphocytes. J Hematol Oncol (2021) 14:21. doi: 10.1186/s13045-020-01024-8 33514401 PMC7844995

[31] Brown CE Alizadeh D Starr R Weng L Wagner JR Naranjo A. Regression of glioblastoma after chimeric antigen receptor T-cell therapy. N Engl J Med (2016) 375:2561–9. doi: 10.1056/NEJMoa1610497 PMC5390684 28029927

