# Supplementary figures and images for: Case report: B7-H3 CAR-T therapy partially controls tumor growth in a basal cell carcinoma patient
Source: Front Oncol. 2022 Aug 17;12:956593. doi: 10.3389/fonc.2022.956593 (PMC9428555; doi:10.3389/fonc.2022.956593)

Supplemental Figure 1

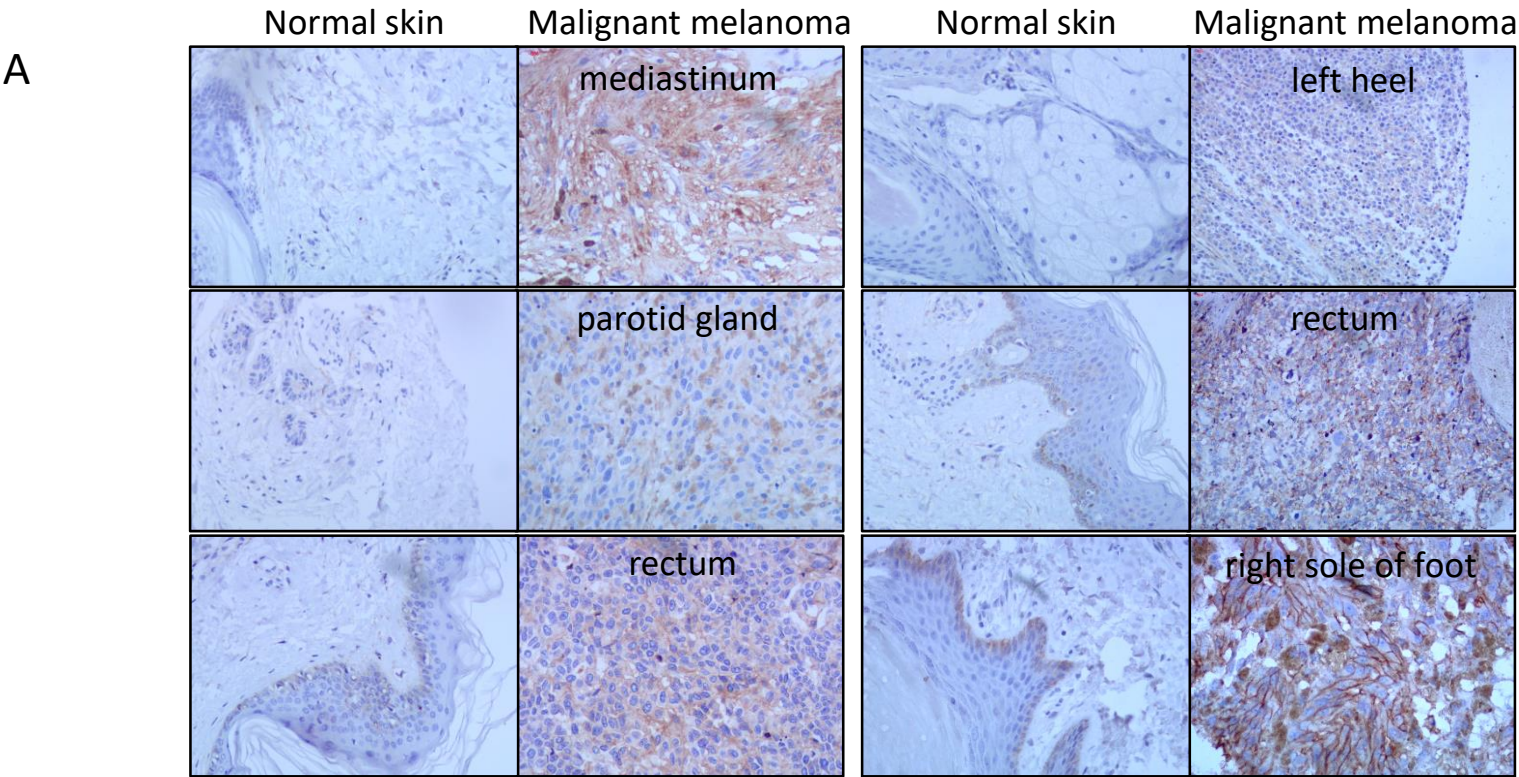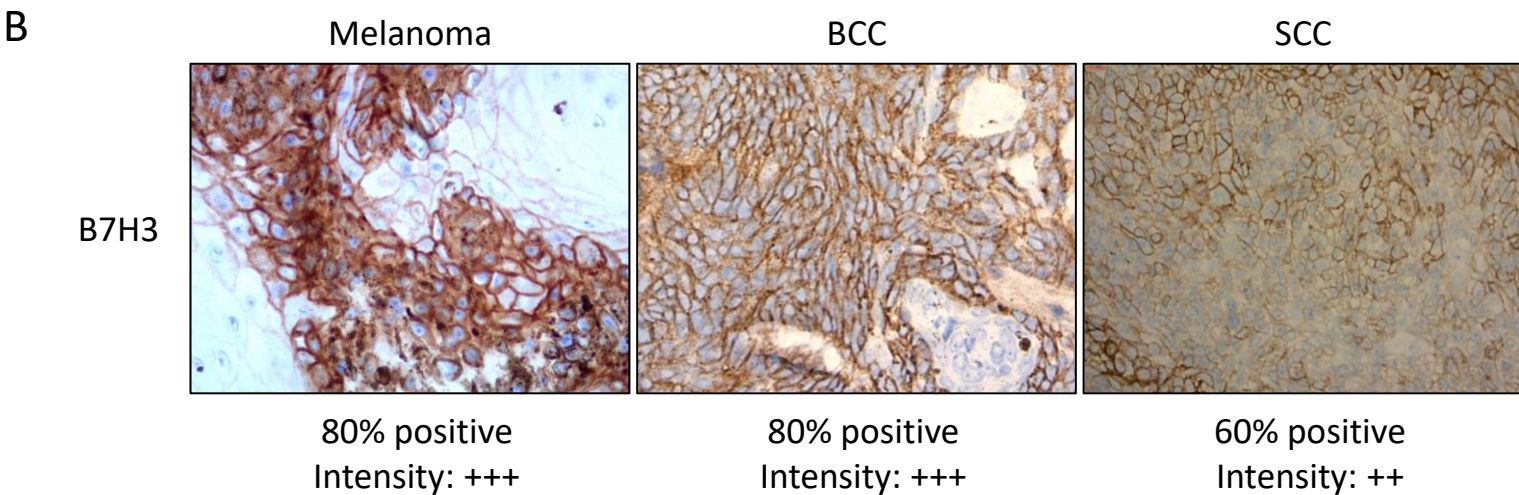

Supplement: Supplementary Figure 1 — Immunohistochemistry detection of B7-H3 expression in skin tumors. (A) B7-H3 expression in malignant melanoma with skin tissue array (DC-MaL11011; 24 cases/24 cores). (B) High expression levels of B7-H3 were observed in melanoma, basal cell carcinoma (BCC), and squamous cell carcinoma (SCC). All IHC slides were analyzed and scored by two individual pathologists independently. [file Image_1.pdf]

Supplemental Figure 2

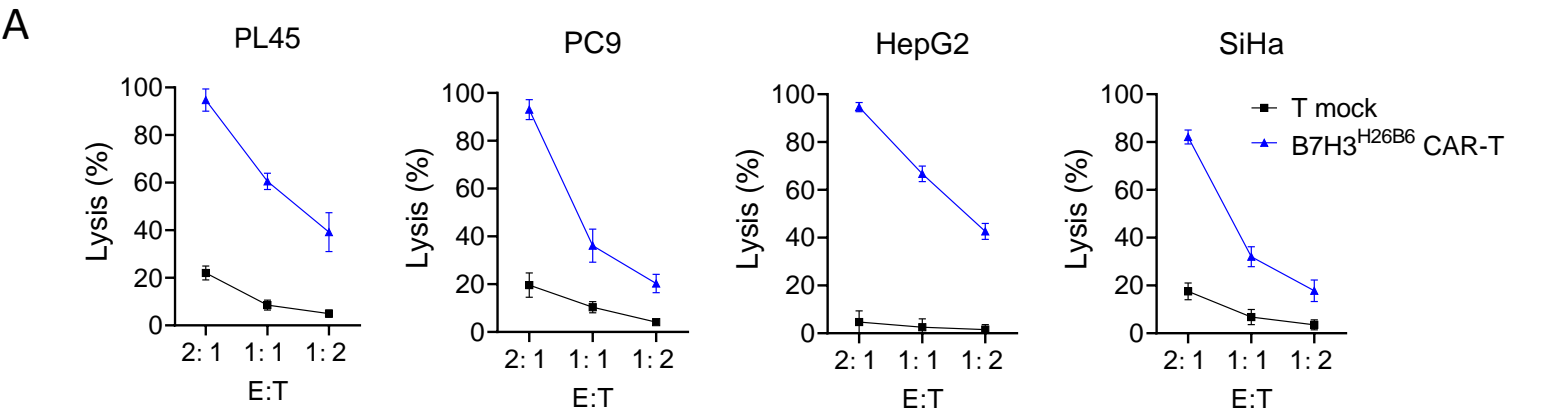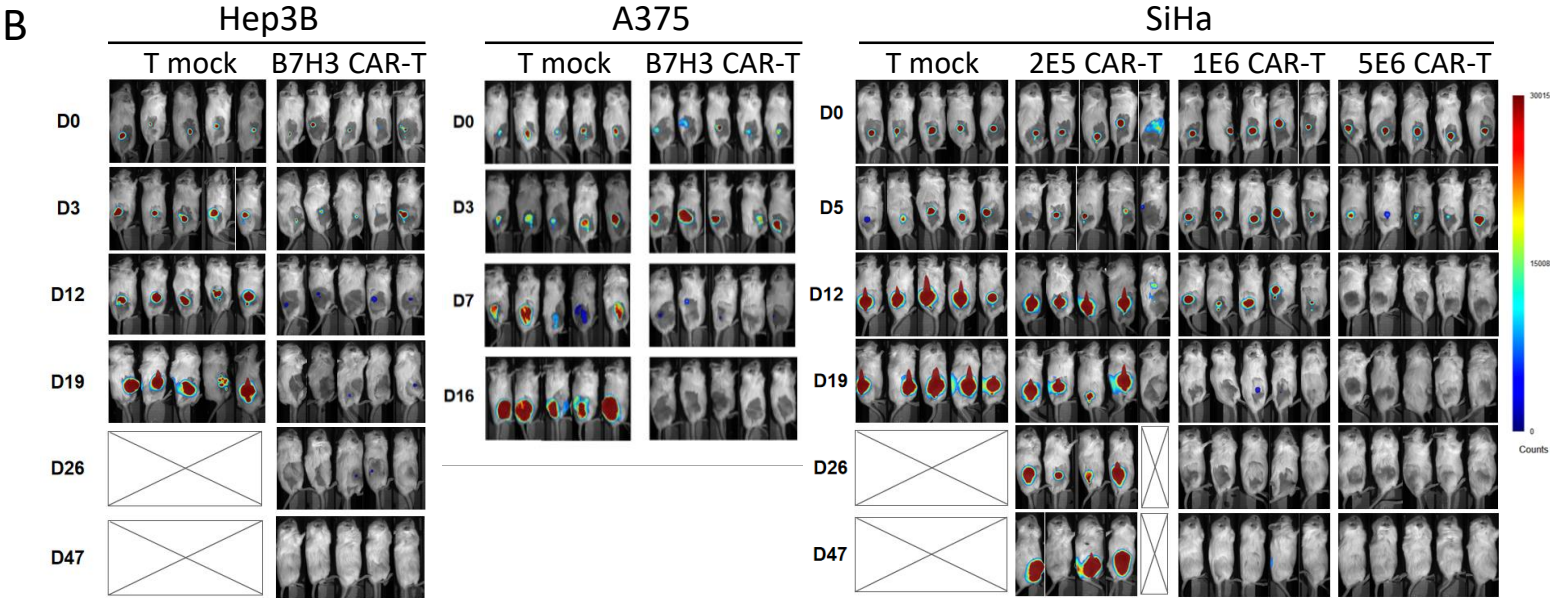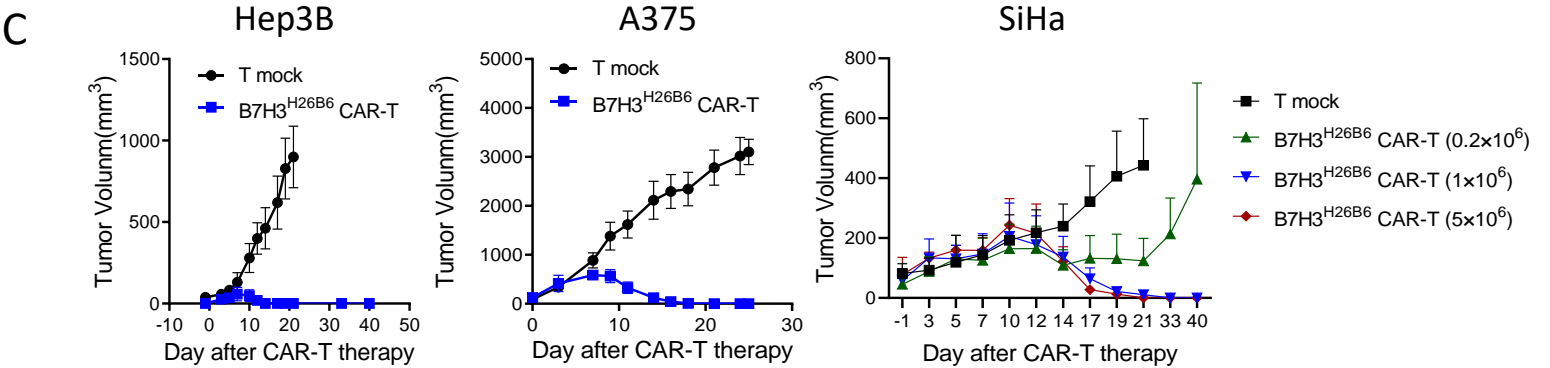

Supplement: Supplementary Figure 2 — B7-H3-CAR-T exhibited excellent antitumor efficacy in vitro and in vivo. (A) B7-H3-CAR-T was effective in killing a variety of solid tumor cells in vitro in a dose-dependent manner. PL45, human pancreatic cancer cells; PC9, human non-small cell lung cancer cells; HepG2, human hepatocellular carcinoma cells; SiHa, human cervical cancer cells. (B) B7-H3-CAR-T effectively cleared transplanted tumors in immuno-deficient NSG mice. Tumor cells (Hep3B, A375 and SiHa) carrying luciferase gene were inoculated subcutaneously on the back of mice for more than 10 days, and the mice were then injected intravenously with B7-H3-CAR-T cells (3×10^6^ cells/mouse were given in mice bearing Hep3B and A375, three therapeutic doses were given in SiHa transplanted mice). (C) Tumor volumes were measured with a vernier caliper, and the results showed that B7-H3 CAR-T completely eliminated the transplanted tumors in mice. [file Image_2.pdf]

Supplemental Figure 4

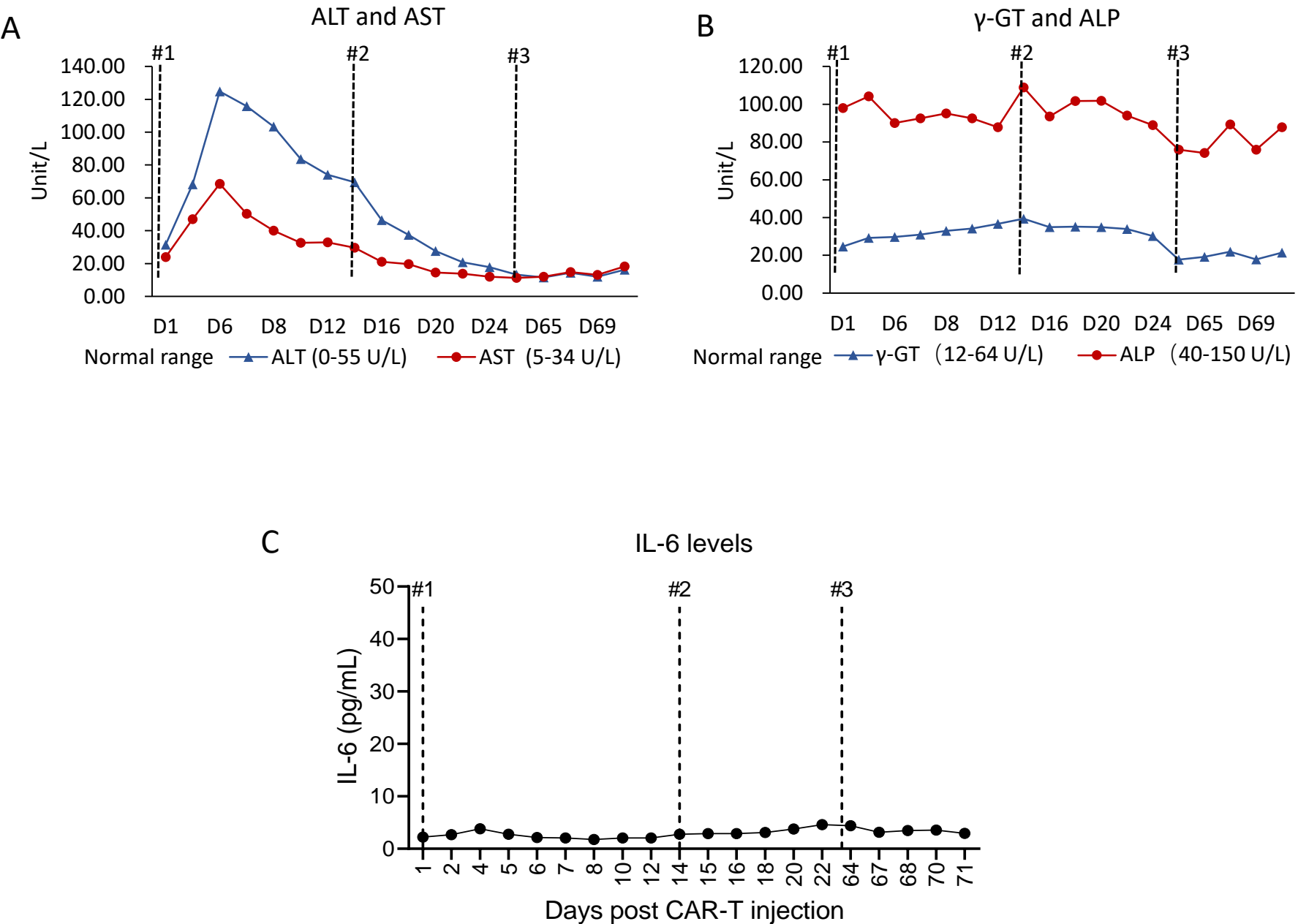

Supplement: Supplementary Figure 4 — Monitoring of liver damage and IL6 levels in blood during immunotherapy. (A, B) The levels of alanine transaminase (ALT), aspartate transaminase (AST), alkaline phosphatase (ALP), and gamma-glutamyl transferase (γ-GT) were examined to evaluate the liver function after CAR-T cell injections. (C) Levels of IL-6 remained low after CAR-T cell injections. [file Image_4.pdf]

Supplemental Figure 5

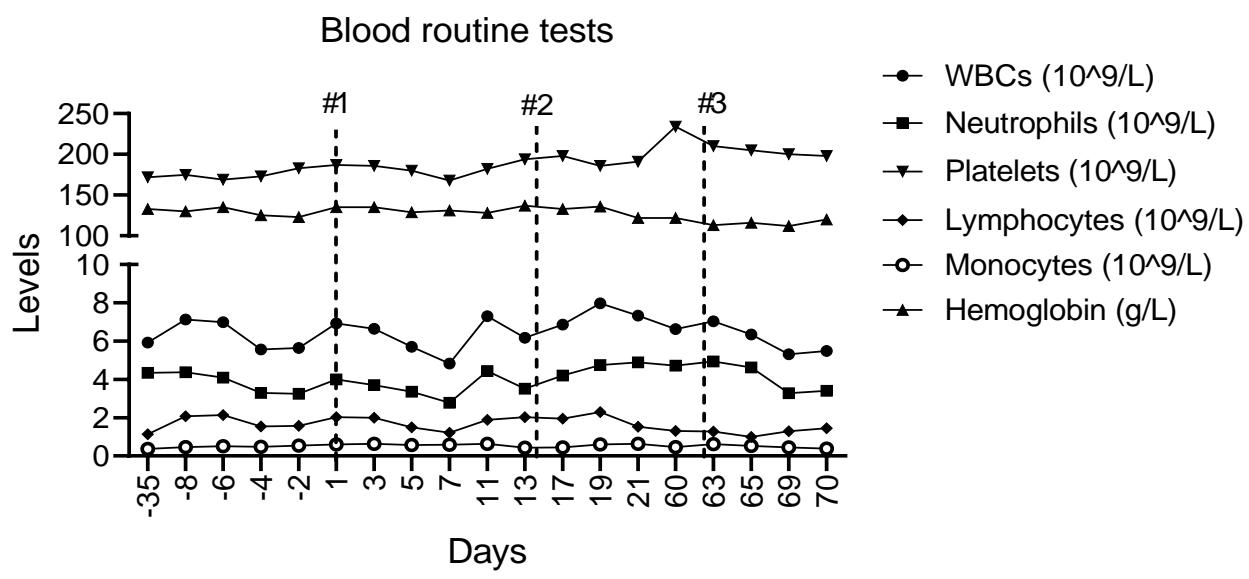

Supplement: Supplementary Figure 5 — Measurement of blood components before and after the CAR-T treatment. [file Image_5.pdf]
