# Supplementary material for: Case report: B7-H3 CAR-T therapy partially controls tumor growth in a basal cell carcinoma patient
Source: Front Oncol. 2022 Aug 17;12:956593. doi: 10.3389/fonc.2022.956593 (PMC9428555; doi:10.3389/fonc.2022.956593)

Supplemental Figure 3

A

	Total cells	Cell activity	CD3+	CD3+/CD8+	CD3+/CD4+	CD3+/CAR+	VCN	Bacteria /mycoplasma
Batch 1	3.2E8	93.2%	99.81%	22.41%	72.94%	52.50%	1.80	negative
Batch 2	1.23E9	88.4%	99.82%	31.74%	59.95%	42.16%	1.56	negative

B

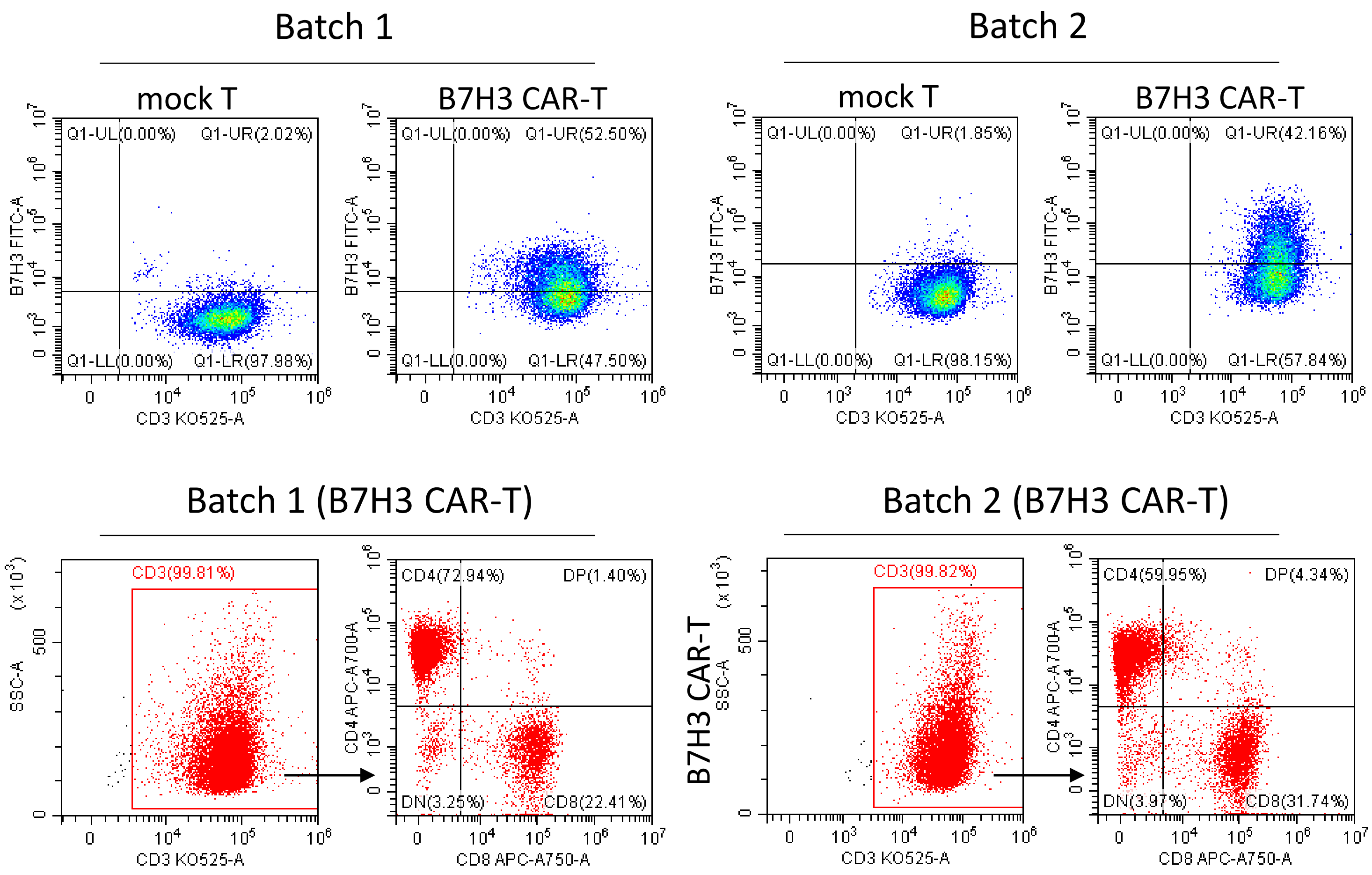

Supplement: Supplementary Figure 3 — Characterizations of the B7-H3-CAR-T cells. (A) The table shows the identification results of two batches of B7-H3-CAR-T cells. CAR-T cell activity was determined by Smart Cell Counter NucleoCounter® NC-200TM (ChemoMetec, Danmark). 200 µL suspended cells was added into Vial-CassetteTM containing dried acridine orange (AO) and DAPI dyes. Dead cells were dyed with AO/PI and counted by NucleoCounter. The cell activity was presented as (total cell number - dead cell number)/total cell number × 100%. A single copy gene (RPPH1)-based duplex qPCR assay was used to determine vector copy numbers (VCNs) in CAR T cell products. (B) Percentages of CD3, CD4, CD8, CAR positive T cells were determined by flow cytometry analysis. [file Image_3.pdf]
